# Delayed presentation of Wilkie’s syndrome after scoliotic curve correction surgery: a case report

**DOI:** 10.1186/s12891-024-07462-6

**Published:** 2024-04-24

**Authors:** Tushar Rathod, Yash Prakash Ved, Deepika Jain, Altamash Patel

**Affiliations:** 1 Department of Orthopaedics, Seth G. S. Medical College and K. E. M. Hospital, Mumbai, Maharashtra India; 2 Department of Orthopaedics, Lokmanya Tilak Municipal Medical College and General Hospital, Mumbai, Maharashtra India; 3 Department of Orthopaedics, Senior Resident Seth G. S. Medical College and K. E. M. Hospital, Mumbai, Maharashtra India

**Keywords:** Aorto-mesenteric angle, Aorto-mesenteric distance, Posterior spinal fusion, Congenital scoliosis, Superior mesenteric artery syndrome, Intestinal obstruction, Acute spinal lengthening, Deformity correction

## Abstract

**Background:**

Superior mesenteric artery (SMA) syndrome, also known as Wilkie’s syndrome, is a rare but serious complication following scoliosis correction surgery. It occurs as a result of mechanical compression of third part of duodenum between the SMA and aorta. This condition occurs most commonly in significantly underweight patients with deformities, and usually during the first week following spinal deformity corrective surgeries. The angle between the abdominal aorta and the SMA gets reduced following spinal lengthening during deformity correction surgery causing compression of third part of duodenum resulting in development of SMA syndrome.

Case presentation.

We present a case of 17-year-old male with congenital scoliosis with a 70-degree scoliotic curve who underwent spinal deformity correction surgery with posterior instrumented fusion. Post-operative course was uneventful and the patient was discharged after suture removal on post-operative day 15. The patient presented after 21-days of symptom onset on post-operative-day 51, with a 3 week history of post-prandial vomiting, abdominal pain and distension which resulted in rapid weight loss of 11 kg. A CT-angiogram showed obstruction at third part of duodenum. After reviewing clinical and radiological profile of the patient, a diagnosis of SMA syndrome was made. Conservative management was tried, but due to rapid deterioration of patient condition and symptoms of complete intestinal obstruction, the patient was treated surgically by gastro-jejunostomy and side-to-side jejuno-jejunostomy, which improved his condition.

**Conclusion:**

SMA syndrome can occur much later than previously reported cases and with potentially life-threatening symptoms following scoliosis correction. Having a high index of suspicion, early recognition of condition and institution of appropriate treatment are essential to prevent occurrence of severe complications including risk of intestinal perforation and mortality. This case highlights management of delayed onset of SMA syndrome, with presentation further delayed after symptom onset, as is common in developing parts of the world, due to limited availability and accessibility of resources, and low socio-economic status of large segments of the population.

## Introduction

SMA syndrome is one of the rare complications of scoliosis correction surgery. It occurs due to vascular compression of third part of duodenum between SMA and abdominal aorta when duodenum traverses in the axilla of SMA [1–4]. The incidence of SMA syndrome was reported to be from 0.013–4.7% [5]. Accurate diagnosis may pose a challenge, and if delayed, may lead to complete intestinal obstruction, for which emergency laparotomy may be required in order to salvage the patient. Mortality rate of up to 33% has been reported in such severe presentations [6]. With regards to SMA syndrome seen after deformity correrction surgeries, the mechanism involved is the reduction in the angle between the two vessels. The aorto-mesenteric angle ranges from 38–65 degrees and is occupied by mesenteric fat pad [7], with the aortomesenteric distance being 10-28 mm.

Conditions predisposing to SMA syndrome are vertebral lengthening after scoliosis correction, cast immobilization of spine in patient with decreased mesenteric fat (for example- underweight patients), considerable weight loss (for example- in malignancies) [8–12]. Corrective techniques in scoliosis result in significant lengthening of vertebral column and an extrinsic compression of distal duodenum as it passes through the sharp angle formed by aorta and spine posteriorly and the SMA anteriorly. Following scoliosis surgery this condition usually develops during first post-operative week [5, 13, 14]. We present a case of congenital scoliosis who underwent deformity correction and fusion with posterior spinal instrumentation, who had symptom onset at 4 weeks post-operatively, but presented to us with severe SMA syndrome, 7 weeks following surgery, which is a late presentation of SMA syndrome.

### Case presentation

A 17-year-old male with non-contributory past history presented to our spine outpatient department with a deformity in the back noticed by the parents 2 years ago, which progressed gradually. Neurological status of the patient was normal.

**Fig. 1:**
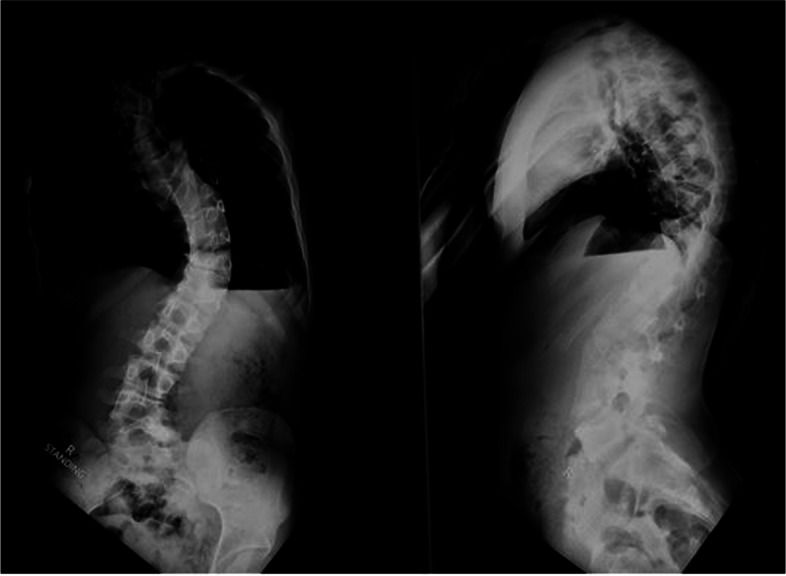
Pre-operative standing, full length x-ray for scoliotic curve assessment. Cobb’s angle of 70 degrees was seen due to congenital scoliosis with D3, D7, D9 and D12 hemivertebra and fusion of right sided posterior element of D3 and D4 vertebra

**Fig. 2:**
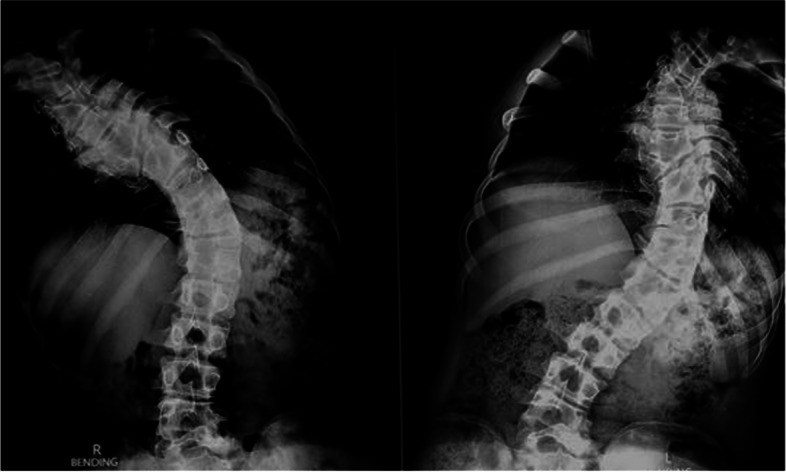
Pre-operative standing, full length lateral bending xray for scoliotic curve assessment showing a stiff curve not showing significant correction by bending, implying a stiff congenital curve

**Fig. 3:**
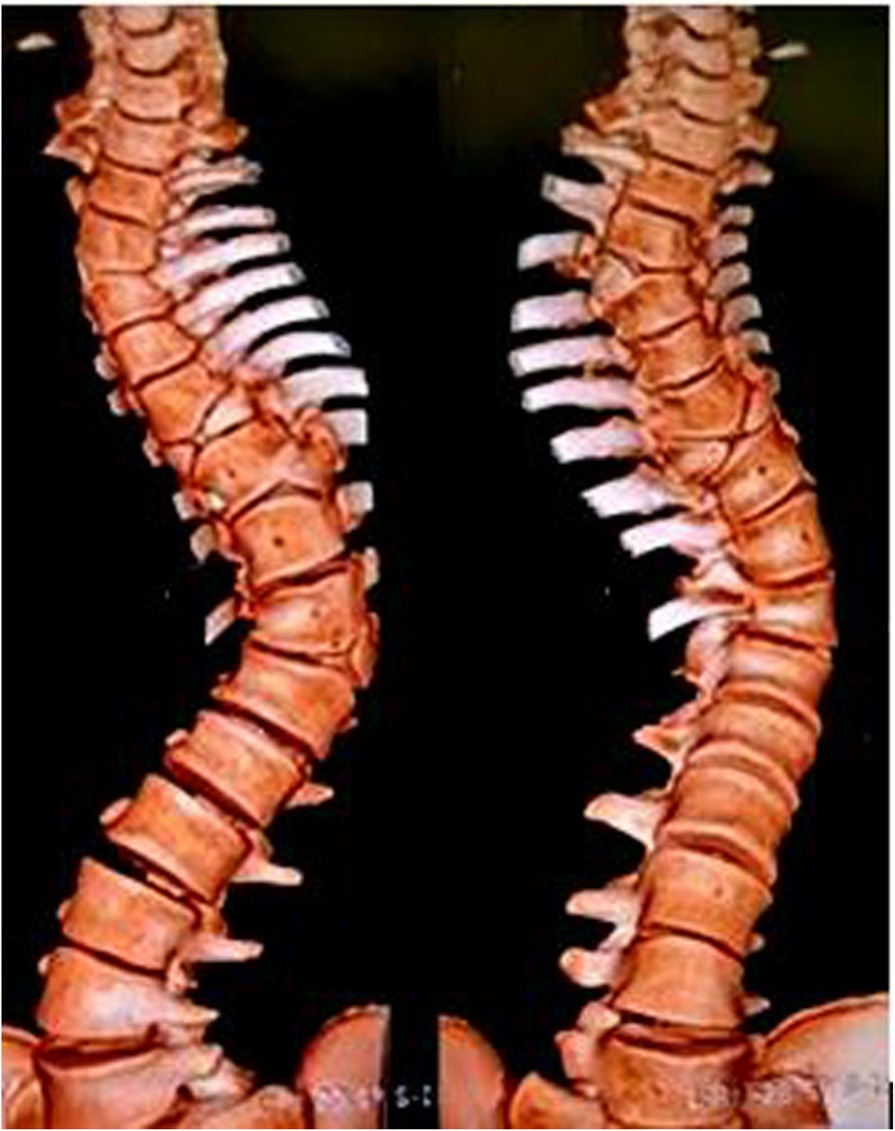
Pre-operative 3D reconstruction of CT scan showing the curve and the congenital disorder of segmentation in the form of hemivertebrae, butterfly vertebra. 3-dimensional anatomy of the curve is better appreciated

On clinical examination left sided thoracolumbar curve was seen with left shoulder elevation, truncal shift to the left, left rib cage prominence posteriorly, which became more obvious on Adam’s forward bending test. There was left flank asymmetry, absence of neuro-cutaneous markers with no neuro-deficit. His weight was 51 kg and height measured 170 cm, with BMI being 17.64. Radiological evaluation shows left sided thoracolumbar curve with 70 degree Cobb’s angle with apex at D12 vertebra with D3, D7, D9 and D12 hemi-vertebra with fusion of right sided posterior element of D3 and D4 vertebra (Fig. 1, 2, 3).

**Fig. 4:**
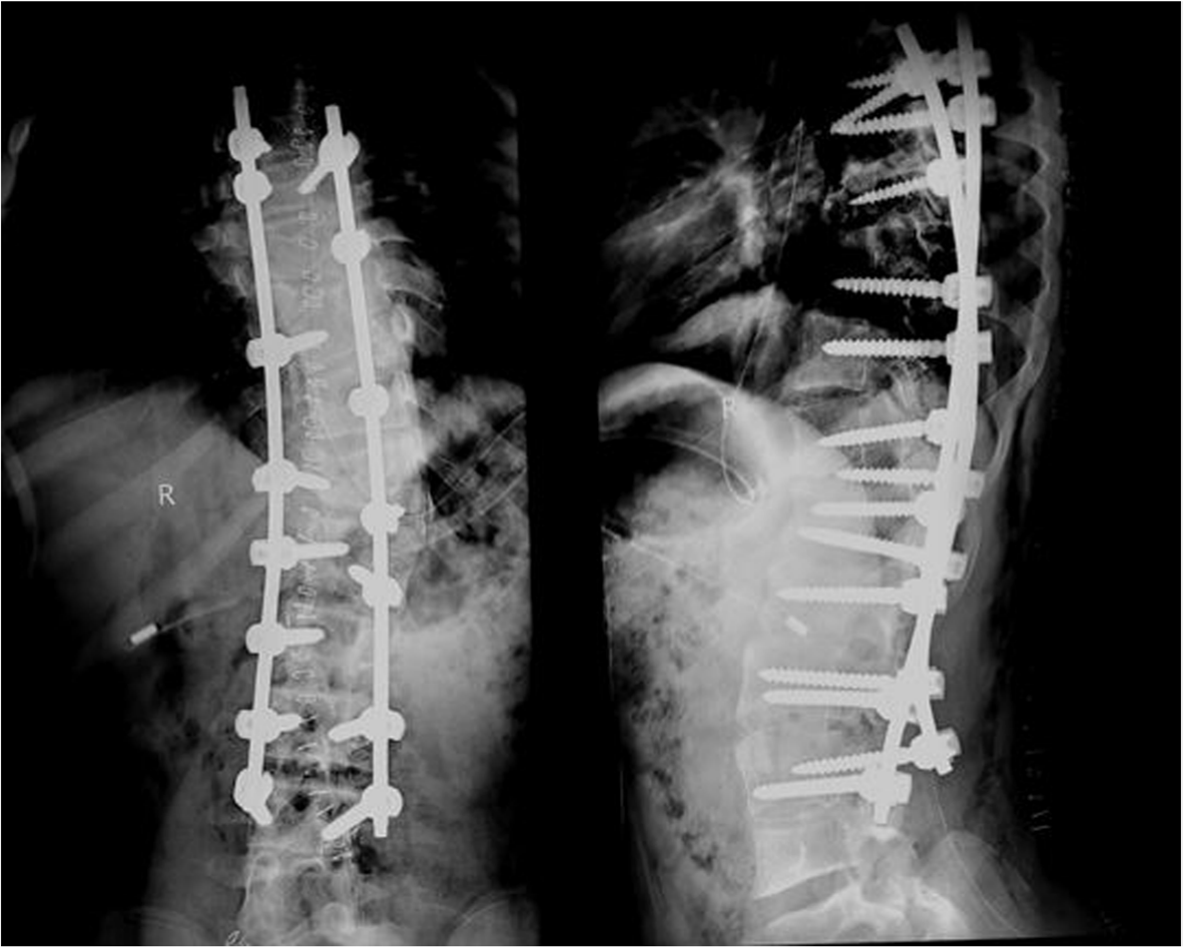
Immediate post-operative radiograph after deformity correction and instrumented fusion from D4 to L4 levels, showing good curve correction of 68.5% from pre-operative measurements. Posterior instrumented fusion with contoured rods and pedicle screws are visible

**Fig. 5:**
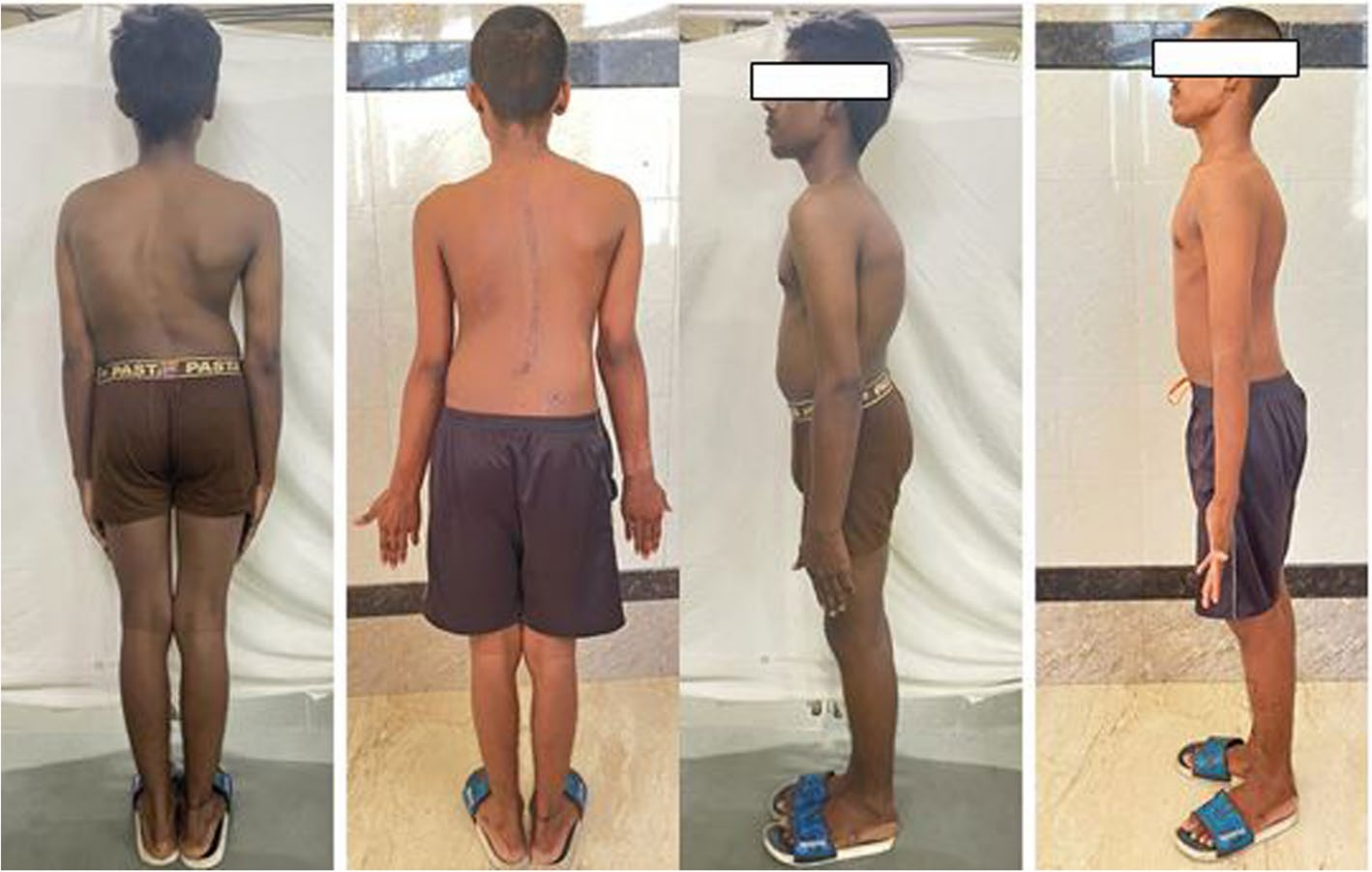
Comparison of pre-operative and post-operative standing image showing the correction and corrected sagittal and coronal balance. The trunk arm distance, the position of the head, level of the shoulder have all been corrected

Preoperative evaluation was done and patient underwent scoliosis correction with posterior instrumented fusion from D4 to L4. Post-operative Cobb’s angle was 22 degree (Fig. 4) after correction (68.5% curve-correction) and had well-balanced spine in sagittal and coronal plane (Fig. 5). Post-operative course was uneventful and patient was discharged after suture removal on postoperative day 15. Patient had episodes of vomiting after consumption of food on post-operative day 30. There was no resolution of vomiting with antiemetic medication prescribed by local healthcare provider. Patient was brought to the hospital on post-operative day 51 with multiple episodes of vomiting, abdominal pain and distension. At the time of presentation to hospital, weight of the patient was 40 kg, due to weight loss of 11 kg over 3 weeks.

**Fig. 6:**
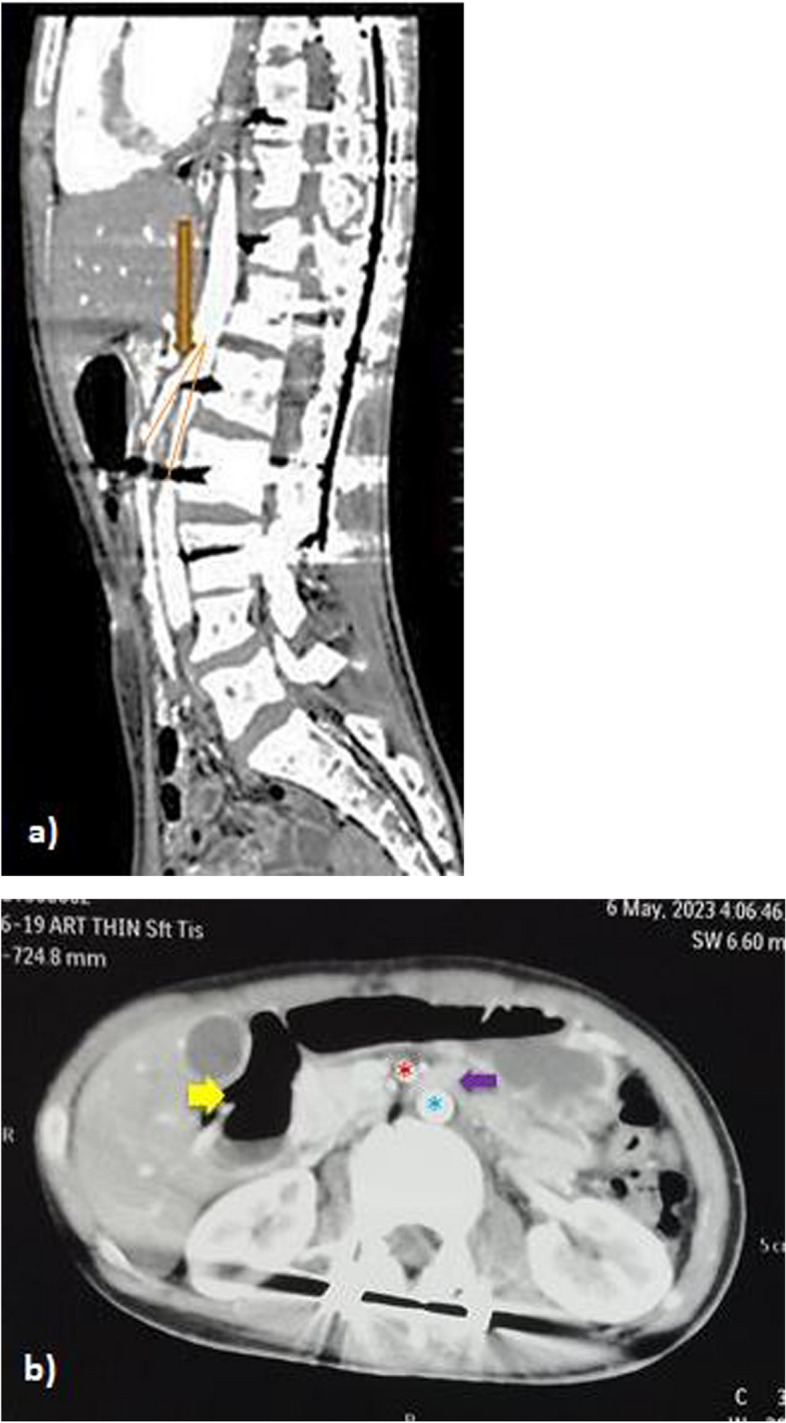
CT angiogram showing reduction in aorto-mesenteric distance and aorto-mesenteric angle with dilated proximal duodenum and collapsed duodenum distal to the compression site between the aorta and SMA. **a** Arrow points to the angle formed between the abdominal aorta and its branch, the Superior Mesenteric artery. It is reduced to 20 degrees (normal – 38–65 degrees). This causes an extrinsic vascular compression over the third part of duodenum causing symptoms of intestinal obstruction. **b** Yellow arrow points to the dilated proximal duodenum, Red asterisk is the SMA, Green asterisk is the abdominal aorta, Purple arrow is the collapsed distal duodenum. Aorto-mesenteric distance is 4.4 mm (normal 10-28 mm)

He was further evaluated by ultrasonography and computed tomographic angiography which showed intestinal obstruction with greatly distended stomach with first and second part of the duodenum, with collapsed third part of duodenum. CT angiography showed decreased aorto-mesenteric angle of 20 degrees (normal-38–65 degrees) along with reduced aorto-mesenteric distance of 4.4 mm (normal-10–28 mm) which confirmed diagnosis of SMA syndrome (Fig. 6a and 6b).

Medical therapy with correction of dyselectrolemia, nasogastric tube decompression and intravenous hydration, complemented with nutritional support, was initiated. The patient continued to deteriorate on medical therapy. In view of a delayed presentation, severity of symptoms, and a picture of long-standing, gradually progressive symptom complex, the patient was treated surgically by exploratory laparotomy and gastro-jejunostomy with side-to-side jejuno-jejunostomy on post-operative day 54. Intraoperatively, a grossly dilated proximal duodenum and stomach was found, and distal part of the duodenum was collapsed beyond the compression caused between the SMA and the aorta. No other significant cause of obstruction was found. Postoperative course was uneventful, and the patient made complete recovery. He was discharged 14 days after surgery. Patient is doing well at 1-year follow up with maintained spinal correction, and no recurrence of gastrointestinal symptoms or complication os surgery.

## Discussion

SMA Syndrome is defined as prolonged postoperative nausea and vomiting for more than one week associated with an ileus, requiring supplemental nutrition coupled with radiological confirmation of constriction of third part of duodenum and delayed gastric emptying [15].

Symptoms of SMA syndrome usually occur after five to seven days of scoliosis surgery [5, 6, 13, 14, 16]. Patients often present with persistent bilious or non-bilious vomiting along with abdominal distension and epigastric tenderness. Post-operative paralytic ileus is a close differential, which occurs secondary to general anaesthesia, to electrolyte imbalance or to opioids for pain. Delayed onset of persistent recurrent vomiting following scoliosis correction surgery should raise suspicion of SMA syndrome especially in high risk patients, as opposed to paralytic ileus, which is usually seen immediately post-operatively [17], within a few hours to 1–2 days postoperatively and spontaneously resolves in 3–5 days [14].

At risk are those patients who underwent scoliosis surgery, had a staged procedure, a lumbar modifier of B or C as per Lenke classification, a low preoperative BMI of less than 18 [18], or patients having weight percentile for height of 5% [19]. and increased stiffness of a thoracic scoliosis [5, 20]. height > 50%, weight < 25% percentile, BMI < 25th percentile, sagittal kyphosis, increased thoracic rigidity and acute spinal lengthening. It is to be noted that the degree of scoliotic deformity correction was not significantly correlated to the development of SMA syndrome [14, 21, 22]. Our patient had a BMI of 17.64, which falls under the at-risk category. Low weight for height indirectly translates to reduced amount of body fat, including mesenteric fat.

In our case patient developed recurrent vomiting with abdominal distension on postoperative-day 30, which was managed with some anti-emetic medication by the local healthcare provider, without any improvement. Patient presented to our institute 3 weeks post-symptom onset. The delay in seeking proper healthcare for disease is commonly seen in developing parts of the world, often leading to challenges that would otherwise be possible to avoid via timely intervention.

SMA syndrome is diagnosed by a battery of tests starting with a plain abdominal X-ray, barium swallow X-ray, computed tomography (CT), abdominal ultrasound (US), magnetic resonance imaging (MRI), endoscopy and endoscopic ultrasonography (EUS) [23].

Most of the cases of SMA syndrome can be managed conservatively in the form of insertion of nasogastric tube, intravenous hydration and correction of electrolyte imbalance – the ‘drip and suck’ approach, along with low volume, high calorie diet [17, 23]. Oral intake should be restricted. A nasojejunal feeding tube must be considered and passed distal to the site of the duodenal obstruction using imaging assistance to provide enteral feedings and achieve gradual weight gain, or if necessary total parenteral nutrition should be given [5]. Medical management may be successful in patients with a short history, moderate symptoms and incomplete duodenal obstruction [24]. Medical management should be tried for a minimum of 6 weeks in appropriate clinical setting [25]. Simple postural changes like knee chest position, left lateral decubitus position and upright position may facilitate decompression. Additional treatment strategies include strengthening of lax abdominal musculature to correct exaggerated lumbar lordosis [15, 26].

Indications for surgical management include failure of medical management for a reasonable period of time, a long interval between symptom onset and presentation, presence of life threatening complications like metabolic alkalosis, electrolyte imbalance and aspiration pneumonia and complete intestinal obstruction [27]. Most of the reported deaths by the condition involve patients in whom the diagnosis was markedly delayed or was completely missed [5]. In our case, early surgical management was proposed for the patient, in view of clinical worsening even after institution of medical therapy, to the point that the patient developed symptoms of frank complete intestinal obstruction. According to a metaanalysis of post-deformity correction SMA syndrome, 73.1% were treated conservatively 26.9% were managed operatively after the conservative treatment failed [44]. A variety of surgical options for failure of conservative management are now available-

- Duodenojejunostomy [28, 29] being the most commonly done procedure, involves constructing an anastomosis to bypass the obstruction in the third part of the duodenum. However, the final decision of type of procedure is up to the surgeons discretion.
- Gastro-jejunostomy [24], which was the operating surgeon’s preference as per his training, was done in this case. However, it is noteworthy that gastrojejunostomy may have increased risk of peptic ulceration and other postoperative complications like blind loop syndrome and recurrence of symptoms due to non-decompression of the duodenum. No such adverse events were seen to occur in this case.
- Ladd procedure [30, 31]- steps of the procedure include mobilization of the Ligament of Treitz, mobilization of the right colon, complete derotation of the duodenum, delivery of the small bowel to the right upper quadrant, and appendectomy
- Strong procedure [3, 24, 32, 33]—Division of the ligament of Treitz with mobilization of the duodenum for caudal displacement. This option has a failure rate of 25%
- Vascular infrarenal transposition of the SMA [34]—safe and feasible surgical option with more physiologically favourable outcomes comparable to gastrointestinal bypasses
- Various modern modalities of performing the above surgeries such as robotic surgery and laparoscopy have been used [32, 35–40]
- Total gastrectomy with oesophago-jejunal anastomosis [41].

A table including reports of surgically managed patients has been included (Table 1).

**Table 1:** List of studies including patients which were required to be managed surgically over 43 years [28–30, 41, 43–50]

Author	Year	Age	BMI/Weight	Days post scoliosis correction	Days from symptom to surgery	Curve	Correction	Surgery
Evarts et al	1971	12	Not available	4	Not available	T4-L1	30	Division of ligament of Trietz
		24	Not available	7	Not available	T7-L1	44	Duodenojejunostomy
Kennedy et al	1983	14	Thin	40	Not available	Thoracic	19	Total gastrectomy with oesophago-jejunal anastomosis
Amy et al	1985	16	Not available	16	Not available	T4-11	Not available	Ladd procedure
Moskovich et al	1986	17	Not available	9	Not available	T5-11	44	Duodenojejunostomy
Crowther et al	2002	15	45 kg	7	32	Right AIS	57	Duodenal– jejunal flexure was fully mobilized, and the jejunum passed behind the SMA to lie on the right
Andrews et al	2005	14	Not available	13	7	AIS	Not available	Duodenum mobilisation from the retroperitonium, transection and re-anastomisosis anterior to the superior mesenteric artery
Trisikos et al	2005	14	34 kg	1	Not available	Not available	44	Open derotation of duodenum and jejenum
Pan et al	2007	12	16.64	2	27	T6-11, T11-L4	35	Gastrojejunostomy
Keskin et al	2014	17	43.5 kg	5	7	AIS 1B		side-to-side duodenojejunostomy
Horn et al	2015	12	18.6	14	Not available	T5-T12 55 R, T12-L4 27 L	30, 7	Stamm gastrostomy
Cullis et al	2016	12	14.7	Immediately	420	Not available	Not available	Laparoscopic duodenojejunostomy
Ovalle-Chao et al	2017	14	13.4	1	Not available	Not available	48	Duodenojejunostomy
Rai et al	2019	13	17.89	6	21	T4-12 115R	Not available	Laparoscopic duodenojejunostomy

SMA arises from aorta at the level of first lumbar vertebra. Third part of duodenum lies at an acute angle ranging from 38 to 65 degrees [42], between the abdominal aorta posteriorly and SMA anteriorly, with the normal distance between them being in the range of 10-28 mm [42]. There is a positive correlation between BMI and both the above mentioned values. Hence, as BMI increases so do both the values, and vice versa. Reduction in this angle can lead to compression of duodenum which can be predisposed by many factors like rapid reduction in body weight, reduced retroperitoneal fat content, or acute spinal lengthening [19, 42]. An aortomesenteric angle below 22 degrees and an aortomesenteric distance lesser than 8 mm is the threshold for the radiological diagnosis of SMA syndrome in the appropriate clinical setting [23]. Scoliosis associated with increased sagittal kyphosis is usually associated with collapse of trunk, and following correction of deformity elongation of trunk becomes more remarkable leading to further narrowing of aorto-mesenteric angle [14]. Acute lengthening of spine during deformity correction significantly contributes to narrowing of aorto-mesenteric angle and hence leading to development of SMA syndrome [22]. Combined risk factors of low preoperative body mass index and rapid postoperative weight loss should raise index of suspicion for early diagnosis of SMA syndrome and starting appropriate management [13].

Our patient developed rapid weight loss of 11 kg post-operatively, bringing the BMI further down to 13.84, and also had acute lengthening of spinal column which predisposed to narrowing of aorto-mesenteric angle to 20 degrees and aortomesenteric distance to 4.4 mm, leading to compression of third part of duodenum and hence, to the development of SMA syndrome.

Onset of SMA syndrome usually starts within 7 days of spinal deformity correction surgery, but late onset disease has been reported in literature ranging from several weeks to 4 years after scoliosis surgery [15, 16, 20, 43]. Onset of symptoms in this case was late, started from post-operative day 30, and diagnosis of SMA syndrome was confirmed when radiological investigations were performed when at the time of presentation of the patient to our institute 3 weeks after symptom onset, on post-operative day 51. Hence, this case reports late presentation of SMA syndrome with further delay in seeking specialist medical care, along with failure of conservative management and patient deterioration, and hence was treated surgically in the form of gastro-jejunostomy with side to side jejuno-jejunostomy.

## Conclusion

Early diagnosis and management are the key factors for successful treatment of SMA syndrome. Most of the patients who undergo early diagnosis can be managed conservatively in the form of nasogastric decompression, electrolyte correction and nutritional support. High index of suspicion will lead to early diagnosis and appropriate conservative management.

The significance of close monitoring of rapid post-operative weight loss and need for early intervention cannot be over-emphasized. We have presented a case of SMA syndrome with late onset and further delay in seeking specialist medical attention, leading to failure of conservative management hence required surgical treatment in the form of gastro-jejunostomy and side to side jejuno-jejunostomy.

If diagnosis of SMA syndrome is missed, it can cause considerable morbidity and result into potentially life threatening complications like intestinal perforation, septicaemia and mortality.

## Data Availability

No datasets were generated or analysed during the current study.

## Abbreviations

SMA: Superior mesenteric artery
CT: Computed Tomography
